# Aphtha

**Published:** 1850-11

**Authors:** 


*Aphtha.* By J. YALE WARE, Mass.—The following simple prescription has proved a specific in my hands in many hundred cases of aphtha. I learnt it of Dr. Eli Ives, of New Haven. R. Ipecac. gr. vi; Tinct. Opii; Es. Meneth. pip. aa. gtt. iv; boiling water xxvi tea-spoonfuls. Sweeten with loaf sugar. Dose, a tea-spoonful every two hours. At the same time apply to the tongue equal parts of a powder of borax and loaf sugar, which the child will carry over the mouth. I have never known the above to fail in any case of infant's sore mouth. It generally cures in two days. Occasionally, in delicate subjects, the disease returns again, when the remedy needs repeating.—*Amer. Jour. Med. Sciences.*

